# Intraocular Pressure Changes After Rhinoplasty: A Before‐and‐After Cross‐Sectional Study

**DOI:** 10.1002/hsr2.70389

**Published:** 2025-01-22

**Authors:** Ali Goljanian Tabrizi, Matin Ghazizadeh, Atiyeh Zahedi

**Affiliations:** ^1^ Department of Otorhinolaryngology, Head and Neck Surgery, Taleghani Hospital Shahid Beheshti University of Medical Sciences Tehran Iran

**Keywords:** ecchymosis, edema, intraocular pressure, rhinoplasty

## Abstract

**Background and Aims:**

Rhinoplasty, a prevalent cosmetic surgery, often involves lateral osteotomy, which can lead to ocular complications such as edema and ecchymosis. A potential complication is increased intraocular pressure (IOP) postoperatively. This study aims to investigate the impact of lateral osteotomy during rhinoplasty on IOP in patients.

**Methods:**

This prospective before‐and‐after cross‐sectional study evaluated the effect of lateral osteotomy during rhinoplasty on IOP in 96 patients. Preoperative ophthalmologic examinations included best‐corrected visual acuity, slit lamp biomicroscopy, Goldmann tonometry, and dilated fundoscopy. IOP measurements were taken three times per eye at baseline, during surgery, on the first postoperative day, after nasal pin removal on the seventh postoperative day, and on the thirtieth postoperative day using a Goldmann tonometer. The mean IOP was recorded at each time point.

**Results:**

The study found no significant changes in IOP at 1, 7, or 30 days postsurgery (*p* = 0.194). Additionally, the type of surgery did not significantly affect ocular internal pressure (*p* = 0.622).

**Conclusion:**

Rhinoplasty with lateral osteotomy appears to be safe concerning IOP. However, monitoring IOP during rhinoplasty is advisable.

## 1 Introduction

Rhinoplasty is among the top five most common cosmetic procedures globally [1] and may even be the most popular worldwide [2]. The surgery can be performed using two primary approaches: open and closed [3]. Regardless of the approach, periorbital ecchymosis and edema are common postoperative outcomes [4]. Lateral osteotomy, whether performed externally or internally, is a significant contributor to postoperative eyelid swelling [5, 6]. This swelling can cause psychological discomfort due to the appearance of a puffy face and nearly closed eyes, which typically takes 10–14 days to subside. Additionally, excessive eyelid ecchymosis and edema, coupled with nasal obstruction, can lead to an unpleasant healing process and potentially permanent skin discoloration [7].

Lateral osteotomy can also result in various intraoperative or postoperative orbital complications [8]. Although rare, complications such as loss of vision due to direct trauma to the globe [9] and transient diplopia have been reported following rhinoplasty [10, 11]. These potential issues raise concerns for both patients and surgeons regarding the condition of a swollen, congested eye after cosmetic facial surgery.

Intraocular pressure (IOP) is a critical indicator of ocular health. Normal IOP is essential for maintaining the eye's structure and function. Changes in the balance between aqueous humor production and drainage can alter IOP. Increased IOP can lead to corneal edema, iris atrophy, cataracts, and optic nerve atrophy. Common symptoms of elevated IOP include blurred vision, epiphora, headache, nausea, vomiting, and a sensation of pressure in the eye [12]. Elevated IOP is a risk factor for open‐angle glaucoma [13, 14], the most prevalent cause of permanent vision loss globally [15]. Fluctuations in IOP are considered an independent, preventable risk factor for glaucoma development, making the monitoring of IOP increasingly important [16]. Prompt diagnosis and management of elevated IOP significantly reduce the risk of visual impairment [17]. These clinical observations suggest that alterations in IOP may occur during the post‐rhinoplasty period.

This study aims to evaluate the impact of lateral osteotomy on IOP in healthy individuals undergoing rhinoplasty.

## 2 Materials and Methods

### 2.1 Patients

The ethics committee of the Shahid Beheshti University of Tehran approved this prospective before‐and‐after cross‐sectional study. All candidates for primary rhinoplasty referred to a teaching hospital's ears, throat, and nose clinic were included for 2 years from 2020. Exclusion criteria were diabetes, hypertension, glaucoma, previous eye injuries, history of eye surgery, and prior use of topical corticosteroid eye drops. Patients with systemic conditions such as carotid‐cavernous fistula and pulmonary hypertension, or those causing changes in episcleral venous pressure, were excluded.

### 2.2 Therapeutic Intervention and Patient Evaluation

All patients underwent a comprehensive preoperative ophthalmologic examination, including best‐corrected visual acuity (BCVA), slit lamp biomicroscopy, Goldmann tonometry, and dilated fundoscopy as baseline data. The IOP of each eye was measured three times, and the mean IOP was recorded. IOP measurements were taken during surgery, on the first postoperative day, after intranasal pin removal on the seventh postoperative day, and on the thirtieth postoperative day using a Goldmann tonometer.

### 2.3 Data Analysis

Quantitative variables were described using mean and standard deviation, while qualitative variables were described using frequency and percentage. One‐way and two‐way repeated measures analysis of variance tests were used to compare IOP across different periods, with a significance level set at 0.05. Data analysis was performed using SPSS software version 24.

## 3 Results

The study group consisted of 60 women and 36 men aged 23–36 years. The demographic and baseline characteristics of the patients are presented in Table 1.

**Table 1:** Baseline characteristics of the patients.

Parameters	Mean ± SD & *N* (%)
Age, years	29.26 ± 3.52
Sex, female	60 (62.5%)
Type of surgery	
External perforation	40 (42%)
Continuous internal	56 (58%)
Existence of edema	60 (62.5%)
Existence of ecchymosis	55 (57%)

Abbreviation: SD, standard deviation.

The average IOPs before and after operations, regardless of the osteotomy approach (external vs. internal), are outlined in Table 2. Although a minimal rise in IOP was detected postoperatively, it was not statistically significant.

**Table 2:** The comparison of intraocular pressure in patients at different times.

	Intraocular pressure (mmHg)	*p*‐value^a^
Before surgery	15.36 ± 2.30	0.194
1 Day after surgery	15.87 ± 1.60	
7 Days after surgery	15.20 ± 2.50	
7 Days after surgery	15.49 ± 2.42	

Abbreviation: ANOVA, analysis of variance.

^a^ Repeated measure ANOVA.

IOP changes considering the surgical approaches (external perforation vs. continuous internal) and the duration since surgery are shown in Table 3. There was no significant interaction effect between the two groups.

**Table 3:** The comparison of intraocular pressure in patients at different times by type of surgery.

	External perforation	Continuous internal	*p*‐value^a^
Before surgery	15.39 ± 2.17	15.32 ± 2.40	0.622
1 Day after surgery	15.81 ± 1.81	15.91 ± 1.45	
7 Days after surgery	14.97 ± 2.04	15.40 ± 2.78	
7 Days after surgery	15.47 ± 2.29	15.49 ± 2.53	

Abbreviation: ANOVA, analysis of variance.

^a^ Repeated measure ANOVA.

## 4 Discussion

Postoperative edema and ecchymosis are common complaints among patients undergoing rhinoplasty. Although these conditions do not cause pain, the resulting deformity and the impact on appearance can lead to discomfort and anxiety [18]. While major orbital complications are rare, there is significant concern about the condition of swollen and edematous eyes postoperatively. Rare orbital complications from inappropriate lateral osteotomy include bleeding, enophthalmos or exophthalmos, periorbital cellulitis, epiphora, and even blindness [19, 20].

The rearrangement and shaping of nasal bones are integral to most rhinoplasty surgeries. Several factors contribute to ecchymosis and postoperative edema, with the type of osteotomy being the most significant. Various osteotomy techniques and drug treatments are recommended to manage these issues [21]. Literature reviews indicate no proven superiority between continuous internal or external perforation techniques of lateral osteotomy in rhinoplasty [5]. Thus, the choice of lateral osteotomy methods among surgeons depends on their experience and comfort [22].

The effect of lateral osteotomy on IOP has been explored in a few studies. In the present study, the mean preoperative IOP increased on the first day after surgery, but this increase was not statistically significant (*p* > 0.05). On the seventh and thirtieth days postoperatively, the IOP gradually decreased but remained higher than the preoperative value (*p* > 0.05).

Dikici et al. [23] evaluated IOP changes before and 1 week after rhinoplasty in 10 patients with Grade 4 periorbital edema and ecchymosis. They found no significant changes in IOP, concluding that lateral osteotomy, if performed correctly, is a safe technique with no ophthalmological risk [23]. The results of Rabie et al.'s [24] study involving 30 patients who underwent rhinoplasty IOP during the 7‐day follow‐up period were consistent with the findings of our study, too. They concluded that rhinoplasty is a safe procedure with no significant changes in ophthalmic physiology [24].

The present study has the advantage of a larger number of cases and a longer postoperative follow‐up period. Also, we compared the effect of two known techniques of lateral osteotomy (continuous internal vs. external perforation techniques).

The results of repeated measures analysis of variance showed that there was no statistically significant difference between the two selected approaches in terms of IOP (*p*‐value < 0.05).

A randomized clinical study was conducted on 84 cases of rhinoplasty to compare the severity of periorbital edema and ecchymosis following external and internal lateral osteotomy. Although they didn't measure the patients' changes in IOP, they reported no significant differences in edema or ecchymosis between external perforating and internal continuous lateral osteotomies [25].

## 5 Conclusion

Both types of lateral osteotomies performed during rhinoplasty have no adverse effects on IOP during the first months after surgery. Despite the unpleasant appearance of peri‐orbital edema and ecchymosis, the procedure is assumed to be harmless. Careful attention should be given to this topic. Nevertheless, we suggest monitoring IOP changes in the first four postoperative weeks, especially in patients with a history of ophthalmologic disease.

## Author Contributions

**Ali Goljanian Tabrizi:** conceptualization, visualization, project administration, supervision, investigation. **Matin Ghazizadeh:** visualization, investigation, project administration, writing–review and editing, supervision. **Atiyeh Zahedi:** data curation, formal analysis, methodology.

## Conflicts of Interest

The authors declare no conflicts of interest.

## Transparency Statement

The lead author Matin Ghazizadeh affirms that this manuscript is an honest, accurate, and transparent account of the study being reported that no important aspects of the study have been omitted, and that any discrepancies from the study as planned (and, if relevant, registered) have been explained.

## Data Availability

The data supporting this study's findings are available from the corresponding author upon reasonable request and in the case of the participant's consent.
